# The effect of environmental contamination on the community structure and fructification of ectomycorrhizal fungi

**DOI:** 10.1002/mbo3.396

**Published:** 2016-08-11

**Authors:** Qibiao Sun, Yaping Liu, Huatao Yuan, Bin Lian

**Affiliations:** ^1^ Jiangsu Key Laboratory for Microbes and Functional GenomicsJiangsu Engineering and Technology Research Center for MicrobiologyCollege of Life SciencesNanjing Normal UniversityNanjingJiangsuChina

**Keywords:** *Cedrus deodara*, community structure, ectomycorrhizal fungi, environmental contamination, fruiting body

## Abstract

Ectomycorrhizal fungi are an essential component of forest ecosystems, most of which can form edible and medical fruiting bodies. Although many studies have focused on the fructification of ectomycorrhizal fungi in phenology, the impact of environmental contamination, especially living garbage, on the formation of fruiting body is still unknown. A field investigation, combined with a high‐throughput sequencing method, was used to study the effect of living garbage pollution on the fructification and hypogeous community structure of ectomycorrhizal fungi symbiosing with cedar (*Cedrus deodara* (Roxb.) G. Don). The results showed that garbage significantly altered soil abiotic and biotic properties, increasing soil urease activity, decreasing the soil exchangeable metal content and phosphatase activity, and ultimately inhibiting the formation of fruiting bodies. The pollution of garbage also changed the community structure of hypogeous ectomycorrhizal fungi where ectomycorrhizal ascomycetes dominated. In unpolluted sites, the relative abundance of ectomycorrhizal ascomycetes and basidiomycetes were almost equal. Although no fruiting bodies were observed in that soil polluted by living garbage, the sequencing result showed that various ectomycorrhizal fungi were present underground, suggesting that these taxonomic fungi had the potential to cope with adverse conditions. This study not only provided a deeper understanding of the relationship between ectomycorrhizal fungal communities and prevailing environmental conditions, but provided a new pathway for the excavation and utilization of the resource of antistress ectomycorrhizal fungi.

## 1 Introduction

Ectomycorrhizal (ECM) fungi are one of the most important participants in nutrient cycling in terrestrial ecosystems, especially for nitrogen, phosphorus, and potassium (Behie & Bidochka, 2014; Cairney, 2011; Nasholm, Kielland, & Ganeteg, 2009; Szuba, 2015). ECM fungi can acquire soil‐insoluble elements by biological weathering and transfer them to their host plants in exchange for plant‐derived carbohydrates. ECM fungi can form symbiotic associations with less than 5% of terrestrial plant species (Landeweert, Hoffland, Finlay, Kuyper, & Van Breemen, 2001), and 28–60% of those plants' photosynthetic products are transferred to the fungi (Cairney, 2012; Fogel & Hunt, 1983; Leake, Donnelly, Saunders, Boddy, & Read, 2001) to form a major source of soil organic carbon. Hence, a stable, and appropriate, ECM fungal community structure can not only accelerate the biogeochemical cycle of soil mineral elements, but maintain the healthy growth of plants and stabilization of its surrounding ecosystem. Moreover, many ECM fungi can form conspicuous mushrooms (Henkel et al., 2011; Natarajan, Senthilarasu, Kumaresan, & Riviere, 2005; Tedersoo, May, & Smith, 2010), with important economic and medicinal value. For instance, truffles, chanterelles, and boletes are delicious wild edible mushrooms that cannot be completely artificial cultivated (Hall, Yun, & Amicucci, 2003); modified amanitin extracted from *Amanita* fruiting bodies has potential medicinal value (Hechler, Kulke, Mueller, Pahl, & Anderl, 2014; Liu et al., 2015). Therefore, the study of artificial cultivation of fruiting bodies, and the ecological function, of ECM fungi has been a focus of attention for ecologists and mycologists.

The continued excessive exploitation of natural resources and the consequent anthropogenic pollution disturbs ecosystem equilibria; however, knowledge of the effect of these human factors on the formation of fruiting bodies and community structure of ECM fungi remains deficient. Thus, it is necessary to study the fructification and community structure of ECM fungi under different environmental conditions, which contributes to the understanding of the effect of environmental disruption on these two aspects.

Cedar (*Cedrus deodara* (Roxb.) G. Don), a widespread greening tree species in Nanjing, can form symbiotic associations with many ECM fungi (Itoo & Reshi, 2014; Jabeen, Ashraf, & Khalid, 2015; Singh & Lakhanpal, 2004). Many cedar trees have been planted on Nanjing Normal University's campus. Many ECM fungi can be observed in the summer and autumn seasons beneath such cedar trees, however, few are seen near the campus avenues adjacent to living garbage dumps, mainly consist of plastics, waste paper and clothes, building wastes, few food residues, etc. So whether ECM fungal mycelia still exist in the rhizosphere of cedar and their community composition changes compared to those rhizospheres unpolluted by living garbage requires further research. Answering these questions will help us to understand fruiting body formation conditions, and it may even contribute to planting activities in polluted areas. Furthermore, investigating the community structure in polluted conditions will provide a feasible approach to restoration in contaminated environments by plants coupled with microbes. We therefore carried out this study on fruiting body formation and community structure of ECM fungi in areas polluted by living garbage and unpolluted environments.

## 2 Materials and Methods

### 2.1 Sample collection

Three cedar trees with an accumulation of garbage in the immediate vicinity were randomly chosen as polluted sites (PS) on 11 September, 2015. Three cedar trees, without garbage, were randomly chosen to represent unpolluted sites (UPS). The trees were at least 200 m apart to ensure statistical independence between sites. At each site, three soil samples were collected from the rhizosphere beneath each cedar tree (about 0.5 m separated each soil sample, samples measured 150 mm × 150 mm to 100 mm depth). A total 18 soil samples were collected and labeled. Then, 18 soil samples were placed in sterile polyethylene bags and brought to the laboratory. All soil samples were sieved (passing a 2 mm square aperture nylon mesh), and some soil samples were air‐dried for the determination of pH, total organic carbon, total nitrogen, and exchangeable element concentration. The remainder soil samples were stored at 4°C for determination of enzyme activity, or at −80°C for ECM fungal DNA extraction.

### 2.2 Determination of soil physical and chemical properties and enzymatic activity

Soil pH was determined in water (ratio 1:2.5 w/v, stirring thoroughly, and settling for 30 min before measurement) (Islam & Weil, 2000). Soil total nitrogen was determined by vario EL III Element Analyzer (Elementar, Germany). Soil organic carbon was measured by TOC Analyser SSM‐5000A (Shimadzu, Japan). Soil exchangeable cations and phosphorus were extracted by the ammonium bicarbonate‐diethylenetriaminepentaacetic acid (AB‐DTPA) multi‐extractant method (Soltanpour, 1985), and the concentration of exchangeable cations were determined by IRIS Intrepid II XSP (Thermo Electron, USA). Soil exchangeable phosphorus was determined by molybdenum blue colorimetry (Murphy & Riley, 1962). The method of Eivazi and Tabatabai (1977) was used to assess soil phosphatase activity. The determination of urease activity was based on the colorimetric determination of the ammonia released after incubation of soil samples with urea solution for 24 hr at 37°C (Alef & Nannipieri, 1995).

### 2.3 DNA extraction and sequencing of ECM fungi

Soil DNA was extracted using a FastDNA^®^ Spin Kit for soil (MP Biomedicals, USA) according to the manufacturer's instructions. According to its concentration, the DNA solution was diluted to 1 ng/μl with sterile deionized water. The primers ITS1‐F(5′‐CTTGGTCATTTAGAGGAAGTAA‐3′) (Gardes & Bruns, 1993) and ITS2(5′‐GCTGCGTTCTTCATCGATGC‐3′) (White, Bruns, Lee, & Taylor, 1990) were used to amplify the internal transcribed spacer 1 (ITS1) of ECM fungal rDNA. To distinguish different samples, barcodes were added to forward and reverse primers to attribute sequences to each sample. All PCR reactions were carried out using a Phusion^®^ High‐Fidelity PCR Master Mix (New England Biolabs, USA). PCR products were electrophoresed on 2% agarose gel. According to the concentration, PCR products were mixed at an equimolar ratio and re‐electrophoresed on 2% agarose gel. Then the target DNA strips were extracted using a QIAquick^®^ Gel Extraction Kit (Qiagen, Germany). Sequencing libraries were generated using TruSeq^®^ DNA PCR‐Free Sample Preparation Kit (Illumina, USA) following manufacturer's recommendations and index codes were added. The library quality was assessed on the Qubit^®^ 2.0 Fluorometer (Thermo Scientific, USA) and Agilent Bioanalyzer 2100 system (Agilent Technologies, USA). Finally, the library was sequenced on an Illumina HiSeq2500 platform by Novogene (Beijing, China, http://www.novogene.cn/) and 250 bp paired‐end reads were generated.

### 2.4 Data processing

Sequenced raw data generated by Illumina were separated by sample according to barcode sequences. Then barcode and primer sequences were removed from each sample. Due to the Illumina Hiseq2500 platform adopting a paired‐end sequencing strategy, reads were merged into a complete sequence as supposed from valid tags. Raw valid tags were produced in FLASH (Magoč & Salzberg, 2011). Merged raw valid tags were filtered in QIIME referring to the default parameters (Bokulich et al., 2013; Caporaso et al., 2010) and high‐quality clean tags were obtained. Chimeric sequences in clean tags were picked out and removed using UCHIME (Edgar, Haas, Clemente, Quince, & Knight, 2011) according to the Unite database (https://unite.ut.ee/) and effective tags were obtained. Effective tags for all samples were clustered into operational taxonomic units (OTU) at 97% similarity: a representative tag with the highest abundance in each OTU was selected for taxonomic assignment by the Uparse algorithm (Edgar, 2013). Taxonomic annotation of representative tags was performed in QIIME by use of the BLAST method (Altschul, Gish, Miller, Myers, & Lipman, 1990), again, with reference to the Unite database (Kõljalg et al., 2013). Finally, to ensure a fair comparison between samples, all samples were normalized to the minimum number of effective tags. Subsequent analyses were based on the normalized data.

### 2.5 Statistical analysis

Rarefaction curves and the α‐diversity indices of Chao1, Shannon, and Simpson were produced in QIIME. For the β‐diversity index, an OTU level‐based dissimilarity Binary‐Ochiai metric (Boyce & Ellison, 2001) was used to measure the pair‐wise community similarity between samples, and principal coordinate analysis (PCoA) was used to visualize the distance matrix of all 18 samples in QIIME. Adonis (Fierer et al., 2010) performed in QIIME was used to test whether, or not, differences among groups of samples were significant. Canonical correspondence analysis (CCA) was performed in R using the Vegan package. *t* test was performed in SPSS 20 (IBM, USA). Heatmaps were generated using HemI1.0 (Deng, Wang, Liu, Cheng, & Xue, 2014).

## 3 Results

### 3.1 Soil physical and chemical properties and enzymatic activities

**Figure 1:**
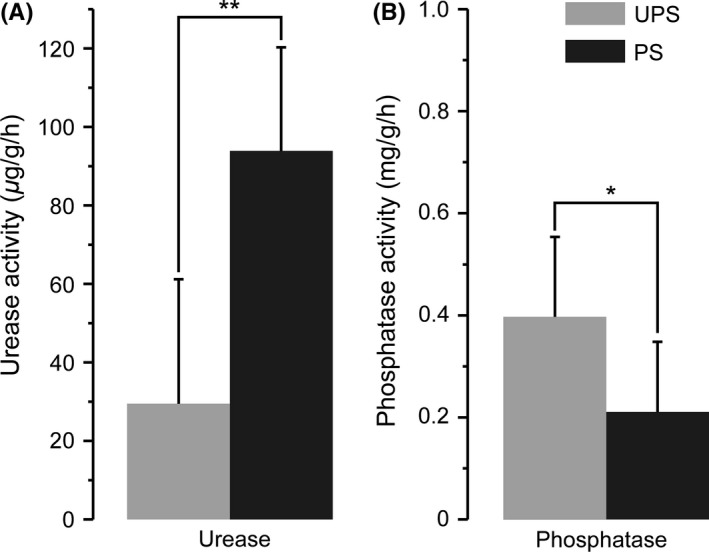
Activities of soil urease and phosphatase. (A) Urease activity is expressed as μg of NH_3_‐N produced through hydrolysis of urea per hr per g of soil at 37°C. (B) Phosphatase activity is expressed as mg of phenol produced per hour per g of soil at 37°C. Values are mean± SD. Asterisks show significant differences identified by *t* test at *p *<* *.05. UPS, unpolluted sites; PS, polluted sites. *p <* *.05, **p <* *.01

Soil pH and the content of exchangeable metals and phosphorus are as listed in Table 1. The soil pH at UPS was significantly lower than that at PS (*t *=* *−2.275, *p *=* *.023). The soil exchangeable calcium, exchangeable iron, exchangeable manganese, exchangeable magnesium, exchangeable nickel, and exchangeable zinc contents at UPS were noticeably higher than those in polluted areas (*t *=* *10.994*, p *<* *.001; *t *=* *2.827*, p *=* *.012; *t *=* *3.650*, p *=* *.003; *t *=* *2.907, *p *=* *.010; *t *=* *2.900, *p *=* *.014; *t *=* *2.305, *p *=* *.035, respectively). The content of soil total organic carbon at UPS was lower than that at PS. The assessment of soil enzymatic activity showed that phosphatase activity at UPS was noticeably higher than that at PS, however, urease activity was lower than that at PS (Fig. 1).

**Table 1:** Soil properties of unpolluted and polluted sites

Sites	pH	TOC	TN	Ca	Cu	Fe	K	Mg	Mn	Ni	P	Zn
		mg/g		mg/kg								
UPS	6.98 ± 0.67	14.65 ± 4.79	1.27 ± 0.45	516.78 ± 0.13	3.11 ± 0.95	63.19 ± 18.42	42.52 ± 13.38	102.28 ± 29.64	41.96 ± 17.24	0.97 ± 0.38	0.42 ± 0.42	6.67 ± 1.75
PS	7.55 ± 0.56	16.36 ± 2.30	0.74 ± 0.12	362.56 ± 30.56	2.73 ± 0.24	43.80 ± 9.17	51.72 ± 16.16	61.75 ± 15.19	23.67 ± 7.69	0.57 ± 0.17	0.48 ± 0.38	4.90 ± 1.49

TOC, total organic carbon; TN, total nitrogen; Ca, exchangeable calcium; Cu, exchangeable copper; Fe, exchangeable iron; K, exchangeable potassium; Mg, exchangeable magnesium; Mn, exchangeable manganese; Ni, exchangeable nickel; P, exchangeable phosphorus; Zn, exchangeable zinc. Values are mean ± standard deviation. UPS, unpolluted sites; PS, polluted sites.

### 3.2 Data analysis

**Figure 2:**
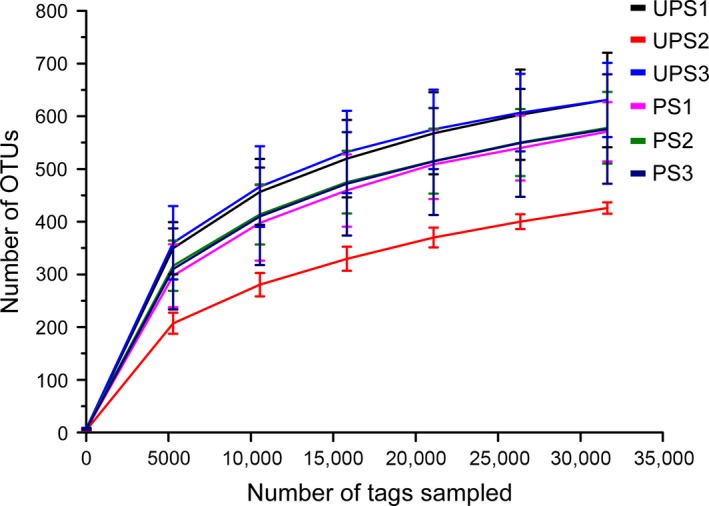
Rarefaction curves of the OTU number at 97% similarity for each sample. Average value of three replicates and error bar are shown. UPS, unpolluted sites; PS, polluted sites.

More than 30,000 effective tags of the ITS1 region were obtained for each sample after merging and quality control. The average length of the effective tags was 260.19 bp. Rarefaction curves of all samples are shown in Figure 2, indicating that sequencing depth can reveal soil fungal community composition. OTUs belonging to the ECM fungal taxonomy (EOTUs) were picked out according to Tedersoo et al. (2010) and the Unite database. Subsequent analyses (α‐ and β‐diversity) were based on selected EOTUs. The α‐diversity indices were calculated as shown in Table 2.

**Table 2:** α‐Diversity indices: all sites

Site	α‐Diversity indices			
	EOTUs	Chao1	Shannon	Simpson
UPS	32.0 ± 6.9	37.4 ± 9.8	4.5 ± 1.1	0.84 ± 0.09
PS	30.0 ± 7.6	34.6 ± 7.7	4.7 ± 0.9	0.85 ± 0.09

Values are mean ± standard deviation. UPS, unpolluted sites; PS, polluted sites.

### 3.3 ECM fungal community structures: all sites

**Figure 3:**
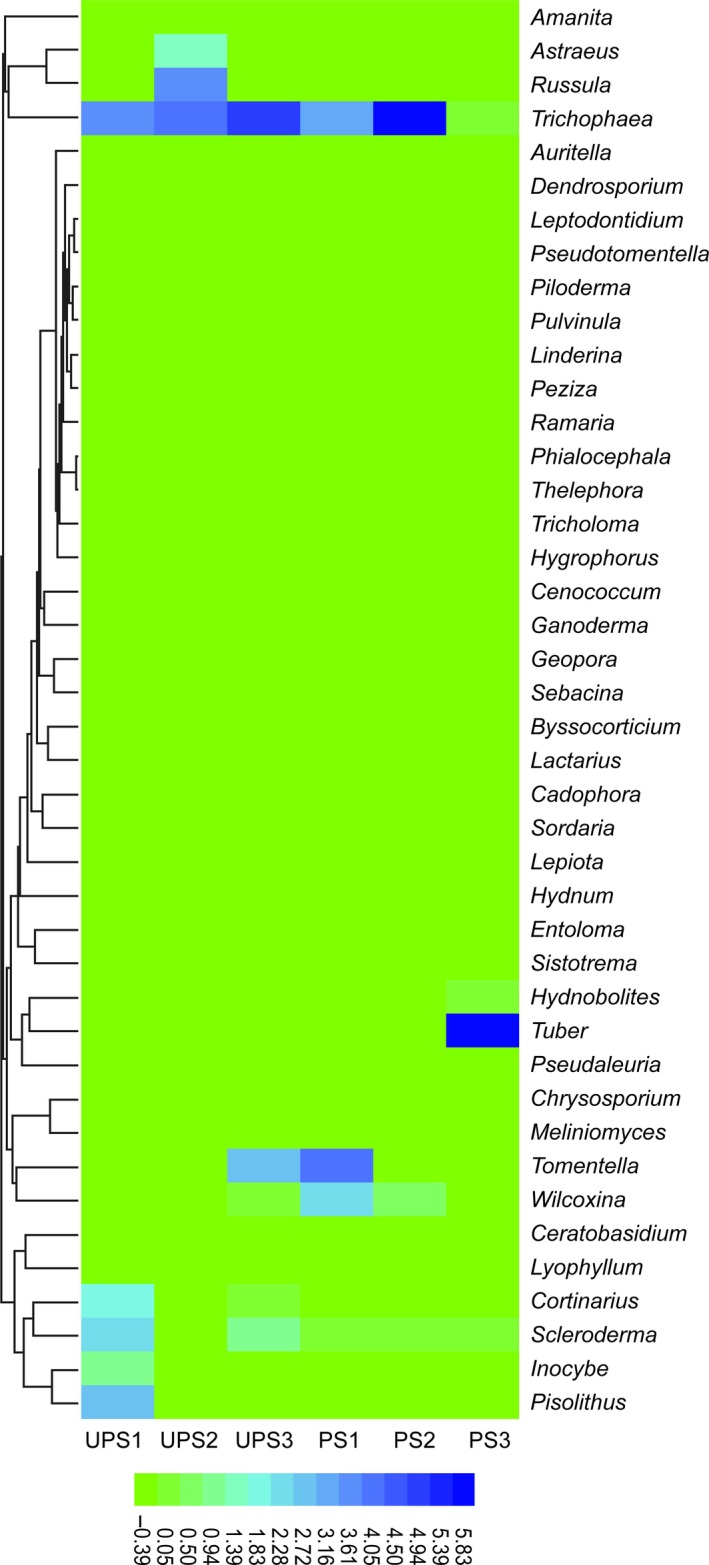
Heatmap of the Ectomycorrhizal (ECM) fungal community structure of all sites at genus level. The highest and lowest abundance of genera are clustered, respectively: the color gradient and similarity reflect the similarity and dissimilarity of ECM fungal community structure in polluted and unpolluted sites. The horizontal axis shows sample information and the vertical axis shows information pertaining to species annotation

All EOTUs were classified into Ascomycota, Basidiomycota, and Zygomycota, due to the negligible number of sequences obtained for Zygomycota, this part of the EOTUs was not analyzed here. At site UPS1, the top five genera were: *Trichophaea*,* Pisolithus*,* Scleroderma*,* Cortinarius*, and *Inocybe*. At site UPS2, the top five genera were as follows: *Trichophaea*,* Russula*,* Astraeus*,* Scleroderma*, and *Cortinarius*. At site UPS3, the top five genera were as follows: *Trichophaea*,* Tomentella*,* Scleroderma*,* Wilcoxina*, and *Cortinarius*. At UPS, most ECM genera were similar, while they were highly dissimilar at PS. At site PS1, the top five genera were as follows: *Tomentella*,* Trichophaea*,* Wilcoxina*,* Scleroderma*, and *Cortinarius*. At site PS2, the top five genera were as follows: *Trichophaea*,* Wilcoxina*,* Scleroderma*,* Russula*, and *Tuber*. At site PS3, the top five genera were as follows: *Tuber*,* Scleroderma*,* Hydnobolites*,* Trichophaea*, and *Pseudaleuria*. A graphical representation, at genus level, is shown in Figure 3.

**Figure 4:**
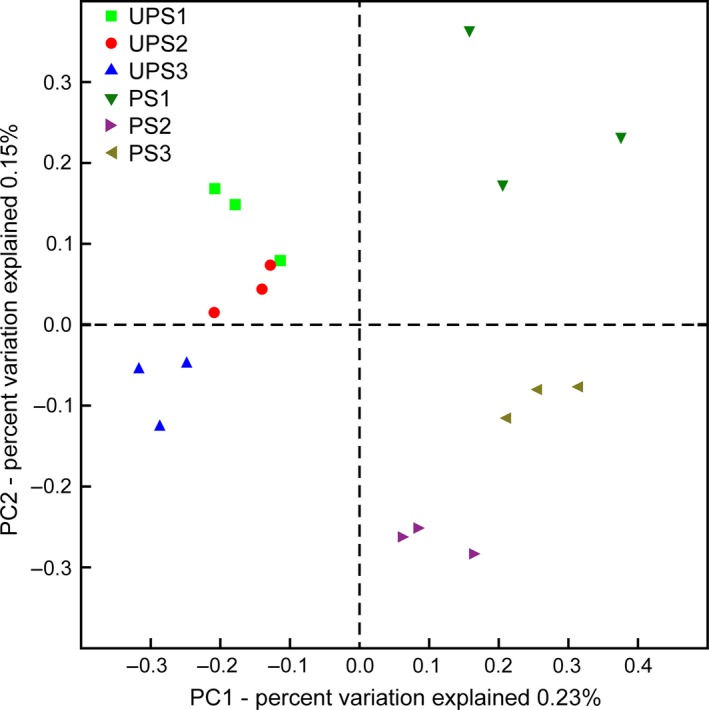
PCoA of community composition at all sites at EOTU level based on richness and relative abundance. The distance between points reflects the differences in Ectomycorrhizal (ECM) fungal community structure in the samples

PCoA were performed to represent the differences in ECM fungal community composition (Fig. 4). The ECM fungal community structures of UPS can be separated from those at PS on PC1 (Adonis *p *=* *1 × 10^−4^, *R* ^2^ = 0.142, number of permutations = 9999). In the polluted group, ECM fungal community structures of different sites were also completely dissimilar (Adonis *p *=* *.003, *R* ^2^ = 0.376, number of permutations = 9999).

### 3.4 Relationship between environmental factors and soil ECM fungal communities

**Figure 5:**
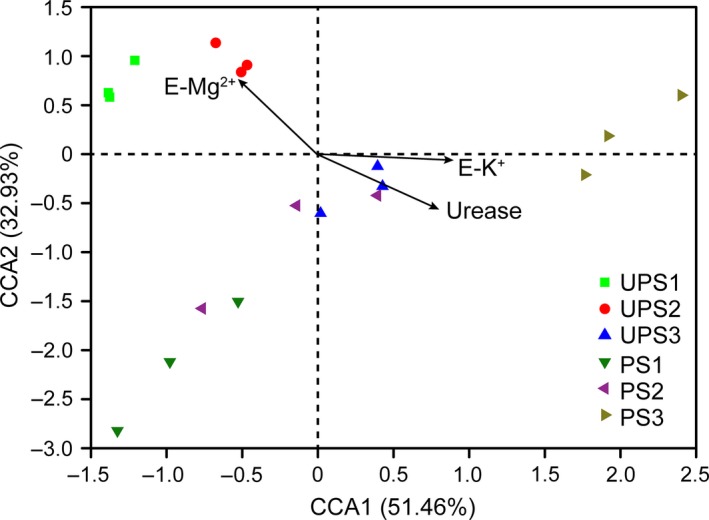
Canonical correspondence analysis (CCA) analysis shows the relationships between Ectomycorrhizal (ECM) fungal community and environmental factors across six sites. The arrows indicate environmental factors, the length of each arrow represents the strength of the relationship between the environmental variable and the distribution of ECM fungi. E‐Mg^2+^, exchangeable Mg^2+^; E‐K^+^, exchangeable K^+^

A subset of environmental parameters (exchangeable K^+^, exchangeable Mg^2+^, and urease) was selected by the BioEnv function of the Vegan package in R: this had the highest Pearson correlation with ECM fungal communities. The selected environmental parameters were used to perform CCA analysis (Fig. 5). The Mantel test indicated a highly significant correlation between environmental variables (exchangeable K^+^ and exchangeable Mg^2+^, and urease activity) and ECM fungal community (*r* = .33, *p* = .004; *r* = .40, *p* = .003; *r* = 0.25, *p* = .017, respectively).

## 4 Discussion

**Figure 6:**
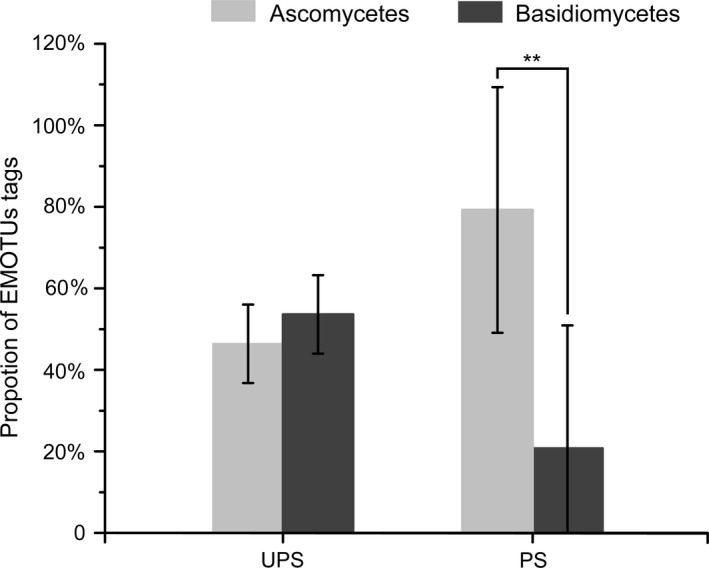
The relative abundance of Ectomycorrhizal (ECM) Ascomycota and Basidiomycota between unpolluted and polluted sites. Asterisks show significant differences identified by *t* test at *p *<* *.05. **p <* *.01

During 3 years of investigation, we observed fruiting bodies of ECM fungi *Calvatia*,* Cortinarius*,* Pisolithus*,* Russula*, and *Scleroderma* at unpolluted sites, although the types of fruiting bodies observed were different each year due to the effects of phenological factors (Barroetaveña, La Manna, & Alonso, 2008; Gange, Gange, Sparks, & Boddy, 2007; Jang & Kim, 2015; Kauserud et al., 2008; Lilleskov, Bruns, Dawson, & Camacho, 2009). However, ECM and even saprophytic mushrooms cannot be observed in garbage‐polluted sites, indicating that living garbage inhibited the epigeous fructification of ECM fungi. Although living garbage inhibited the formation of fruiting bodies, sequencing results still showed many kinds, and at high relative abundance, of ECM fungal mycelia in the rhizosphere of cedar (Fig. 3), suggesting that these taxonomic fungi can survive in contaminated environments but demand more suitable conditions to enable fruiting. In garbage‐polluted sites, ECM fungal species were dominated by ascomycetes (Fig. 6), but ECM basidiomycetes showed fruiting bodies in unpolluted sites albeit at a very low relative abundance of hypogeous mycelia. The result may indicate that living garbage pollution inhibit the formation of ECM fungal fruiting bodies by (1) reducing ECM fungal mycelia biomass which are needed in the early stage of fruiting body formation and (2) altering soil abiotic and biotic factors to inhibit fruiting even although significant numbers of mycelia are present.

Living garbage not only inhibits the formation of ectomycorrhizal mushrooms, but affects the hypogeous ECM fungal community. In living garbage‐polluted sites, the community composition of ECM fungi was dissimilar to that at unpolluted sites and that differed between groups: this may have been caused by the type of garbage (Fig. 4). From sequencing data, it was found that the proportion of ECM Ascomycota and Basidiomycota were similar at unpolluted sites, but ECM Ascomycota predominated at polluted sites (Fig. 6), which indicated ECM basidiomycetes required preferable environmental conditions and ECM ascomycetes can perhaps adapt to various environments in accordance with studies by Grogan et al. (2000), Torres and Honrubia (1997), Visser (1995), and Vralstad, Myhre, and Schumacher (2002) showing that ECM ascomycetes can survive in stressful environments.

The accumulation of living garbage in soil changes soil abiotic and biotic properties. In this study, the soil exchangeable calcium, iron, magnesium, manganese, nickel, and zinc contents and total nitrogen at unpolluted sites were higher than those in polluted sites (Table 1). The decrease in these elements essential to ECM fungal life and activity may be a key abiotic factor affecting ECM fungal fructification; many of these elements can also affect ECM fungal communities. CCA analysis showed that soil exchangeable Mg^2+^ did affect the ECM fungal communities between unpolluted and polluted sites (Fig. 5). These abiotic factors such as metal ions concentration, nitrogen availability, and alkalinity‐induced stresses not only alter ECM fungal community, but affect the formation of fruiting body. In addition, urease activity was not only a key biotic factor inducing change in ECM fungal communities (Fig. 5), but it was also related to the formation of fruiting bodies. The urease activity at polluted sites exceeded that at unpolluted sites (Fig. 1A). Due to soil total nitrogen was lower at polluted sites (Table 1), high urease activity was for accelerating the nitrogen availability in the soil. Furthermore, the uptake of ammonium by EM fungi came at a high carbon cost, which led to less carbon availability for mycelial growth and fruiting body formation (Bidartondo, Ek, Wallander, & Söderström, 2001; Wallander, 1995). Although ECM fungi can exchange nutrients for more plant‐derived carbohydrates (Wallander et al., 2013), when the soil nutrients are insufficient to maintain their own demand, they will supply less nutrients to their hosts and thus obtain less carbohydrates from their host plants so that their biomass declines. However, the hydrolysis of urea would also increase soil pH and the formation of metal carbonates leading to exchangeable metal contents declining (Fujita, Ferris, Lawson, Colwell, & Smith, 2000; Garau, Castaldi, Santona, Deiana, & Melis, 2007; Stocks‐Fischer, Galinat, & Bang, 1999), consequently, reducing mycelial growth and fruiting body formation. Additionally, soil phosphatase activity decreased at polluted sites (Fig. 1B), which mainly because of declined ECM mycelial biomass and that led to lower phosphorus. Phosphorus is required for ECM fungal growth, so a decrease in the amount thereof affected the formation of fruiting bodies. Soil enzyme activities related to soil mineral element mobilization could affect ECM fungi with regard to their mineral element utilization and mycelial growth, thus affecting fruiting and ECM fungal community structure. In summary, soil ECM fungal community structure is not only affected by pH, edaphic enzyme activities, and effective metal content, but is affected by other factors, such as hypogeous saprophytes and host plants (Koide, Fernandez, & Petprakob, 2011).

## 5 Conclusion

The formation of ECM fungal fruiting bodies requires suitable environmental conditions. The pollution of living garbage changes soil abiotic and biotic properties in the rhizosphere of cedar, decreasing mycelial biomass, inhibiting the formation of ECM fungal fruiting bodies, and altering the community structure of ECM fungi. That many ECM fungi detected in polluted sites promoted understanding of ECM fungal fruiting body formation conditions and their environmental adaptability. This research provided a new way of screening and utilizing the resource of antiadversity fungi, which can resist the deficiency of nutrients, even organic and inorganic compounds pollution.

## Funding Information

This work was jointly supported by the National Natural Science Foundation of China (Grant no. 41373078) and a project funded by the Priority Academic Programme Development of Jiangsu Higher Education Institutions (PAPD).

## Conflict of Interest

None declared.
